# 3′-NADP and 3′-NAADP, Two Metabolites Formed by the Bacterial Type III Effector AvrRxo1*[Fn FN3]^♦^

**DOI:** 10.1074/jbc.M116.751297

**Published:** 2016-09-12

**Authors:** Felix Schuebel, Andrea Rocker, Daniel Edelmann, Julia Schessner, Clara Brieke, Anton Meinhart

**Affiliations:** From the Department of Biomolecular Mechanisms, Max Planck Institute for Medical Research, Jahnstrasse 29, 69120 Heidelberg, Germany

**Keywords:** enzyme inhibitor, enzyme mechanism, NAD biosynthesis, second messenger, secretion, toxin, type III secretion system (T3SS)

## Abstract

An arsenal of effector proteins is injected by bacterial pathogens into the host cell or its vicinity to increase virulence. The commonly used top-down approaches inferring the toxic mechanism of individual effector proteins from the host's phenotype are often impeded by multiple targets of different effectors as well as by their pleiotropic effects. Here we describe our bottom-up approach, showing that the bacterial type III effector AvrRxo1 of plant pathogens is an authentic phosphotransferase that produces two novel metabolites by phosphorylating nicotinamide/nicotinic acid adenine dinucleotide at the adenosine 3′-hydroxyl group. Both products of AvrRxo1, 3′-NADP and 3′-nicotinic acid adenine dinucleotide phosphate (3′-NAADP), are substantially different from the ubiquitous co-enzyme 2′-NADP and the calcium mobilizer 2′-NAADP. Interestingly, 3′-NADP and 3′-NAADP have previously been used as inhibitors or signaling molecules but were regarded as “artificial” compounds so far. Our findings now necessitate a shift in thinking about the biological importance of 3′-phosphorylated NAD derivatives.

## Introduction

Bacterial pathogens cause a multitude of severe human, animal, and plant diseases. The vast majority of these bacterial pathogens rely on sophisticated secretion systems by which they either secrete so-called effector molecules into the vicinity of the host cell or translocate them directly into the host cell cytoplasm. All effector molecules, particularly those that are translocated into the host cell cytoplasm, support bacterial invasion, colonization, and proliferation inside the host. They do so by interfering with or remodeling of important host cell processes as for example disguising the invader from the host's immune system (1, 2). For the latter, plant and animal pathogens face entirely different preconditions.

In contrast to animals, plants possess neither a somatic adaptive immune system nor mobile defender cells. Instead, each individual plant cell relies on its innate immunity, enabling it to initiate an appropriate response when infected (3–5). Defense responses of plants against invading microbes are induced at two levels and can eventually culminate in a hypersensitive response (HR)^2^ during which affected cells undergo programmed cell death to prevent systemic spread of the pathogen (6). On the first level, bacterial elicitor active epitopes are recognized by pattern recognition receptors. These elicitor molecules are thus termed pathogen/microbe-associated molecular patterns. Their recognition by specific receptors initializes pathogen-associated molecular pattern-triggered immunity (PTI) (4, 7). Consequently, pathogens evolved effector molecules able to suppress PTI by the host plant and thereby increase microbial pathogenicity (3, 8–10). This elaborate bacterial countermove leads to effector-triggered susceptibility (ETS) of the host and re-establishes microbe pathogenicity.

Certain plant cultivars are capable of overcoming ETS by the second level of defense in which plant resistance proteins specifically recognize one or multiple effectors. Thereby, plants circumvent the pathogen's abrogation of the PTI response and initiate a cellular defense program, leading to effector-triggered immunity (ETI) (10, 11). Effectors recognized by resistance proteins are thus termed avirulence (Avr) proteins.

Most microbial type III effectors (virulent and/or avirulent) are, however, challenging to study because they are unrelated at their sequence or structural level (12–14), making any *a priori* prediction of their functional mechanisms difficult. In addition, most pathogens secrete a number of different effectors, thereby targeting different pathways of their host cell simultaneously (15). Deducing a specific effector function from the observed infection phenotype is consequently almost impossible. Structural studies of these multifaceted effectors have therefore paved the way for follow-up studies focusing on the identification of their biological targets in the host. Such successful pioneering studies were for instance the identification of E3 ubiquitin ligase domains of AvrPtoB and XopL (16, 17) or the inhibitory effect of AvrPto on the Pto kinase activity deduced from the AvrPto-Pto complex structure (18). By a similar approach, the structural homology of the effector protein AvrRxo1 from the pathogen *Xanthomonas oryzae* pv. *oryzicola* to nucleotide kinases led to the recent proposal that AvrRxo1 contains a polynucleotide kinase domain with an unknown toxic mechanism in plants (19).

AvrRxo1 is a type III effector that is highly conserved in various Asian *X. oryzae* pv. *oryzicola* strains (20, 21). It was originally identified as a gene product of this particular pathogen that elicits a non-host HR in maize lines harboring the *Rxo1* resistance gene (20). Interestingly, Rxo1 was also shown to act as a resistance protein in maize against infection by *Paraburkholderia andropogonis* (20), a pathogen encoding a protein highly homologous to AvrRxo1 (NCBI entry ALF40614). *AvrRxo1* and *Rxo1* are therefore most likely involved in a gene-for-gene relationship in certain maize cultivars in which the AvrRxo1 effector is recognized by an ETI program. In contrast, no single gene resistance against *X. oryzae* pv. *oryzicola* has been detected in rice (20). Hence, AvrRxo1 most likely constitutes a virulence factor that elicits ETS, and the rice Rxo1 homolog does not confer immunity (22). The role of AvrRxo1 as a virulence factor is further supported by the finding that it is toxic when expressed in tobacco and yeast cells (20, 21, 23). Furthermore, when ectopically expressed in *Escherichia coli*, AvrRxo1 was shown to inhibit cell growth, a phenotype that could be suppressed by co-expression of AvrRxo2 (19).

The *AvrRxo2* open reading frame (ORF) is part of a bicistronic operon and is found downstream of the *AvrRxo1* ORF in *X. oryzae* pv. *oryzicola* (20). Inhibition of the bacteriostatic phenotype is most likely accomplished by complex formation as inferred from the extensive interaction interface between AvrRxo1 and AvrRxo2 observed in the crystal structure (19). Given that *X. oryzae* pv. *oryzicola* has emerged as a prevalent pathogen that causes rice bacterial leaf streak disease, impairing the production of this staple crop in much of Asia, parts of Africa, and Australia (24), investigating the AvrRxo1/AvrRxo2 system as a major contributor to the pathogen's virulence is overdue.

Here, we describe our bottom-up approach used to identify the effector function of AvrRxo1. We show that AvrRxo1 is a hitherto unknown type of nucleotide kinase that catalyzes the formation of 3′-NADP and 3′-NAADP, two novel compounds that might interfere with conventional NAD(H)/2′-NADP(H)-dependent pathways and host cell Ca^2+^ signaling. In addition to revealing the enzymatic function of AvrRxo1, we show that the associated, chaperone-like protein AvrRxo2 acts as a highly potent inhibitor of AvrRxo1.

## Results

### 

#### 

##### AvrRxo1 Shows Phosphotransferase Activity

**FIGURE 1.**
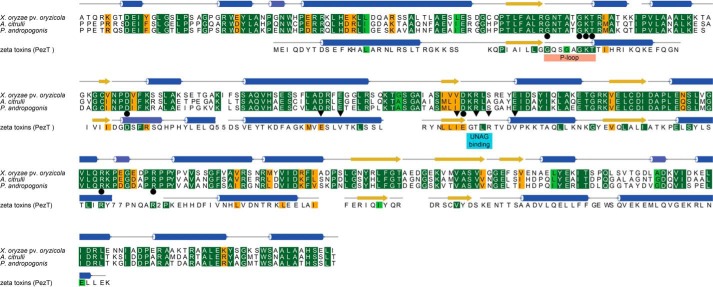
**Structure-based sequence alignment of different AvrRxo1 homologs with zeta toxins.** The C-terminal kinase domain of AvrRxo1 is conserved among *X. oryzae* pv. *oryzicola* (NCBI entry WP_014504815.1) and other plant pathogens including *Acidovorax citrulli* (NCBI entry AIE45656.1) and *P. andropogonis* (NCBI entry ALF40614.1). The primary structure of PezT from *Streptococcus pneumoniae* (NCBI entry WP_000405360.1), a representative UNAG-3P kinase belonging to the ζ toxin family, is given. Residues are colored according to their conservation from *dark green* (high conservation) to *orange* (low conservation). Secondary structure elements are depicted with *cylinders* (α-helices), *arrows* (β-strands), and *gray lines* (loop regions). *Circles* between the sequences indicate residues involved in ATP binding. *Triangles* mark residues of ζ kinases that coordinate UNAG. Other important structural features are highlighted in *boxes*. Note that ATP binding residues, but not UNAG binding residues, are conserved between AvrRxo1 and the ζ toxin PezT.

AvrRxo1 from *X. oryzae* pv. *oryzicola* is a multidomain protein consisting of a central potential kinase domain and an N-terminal domain that has been suggested to contain a thiol protease active site (20, 21). Whereas the kinase domain is conserved among AvrRxo1 homologs from different plant pathogens (Fig. 1), the N-terminal region is highly divergent, and the potential thiol protease active site is only found in a few *Xanthomonas* strains. We therefore exclusively used the truncated variant AvrRxo1ΔN88 that lacks the divergent N terminus in our experiments and will refer to this as AvrRxo1 throughout.

**FIGURE 2.**
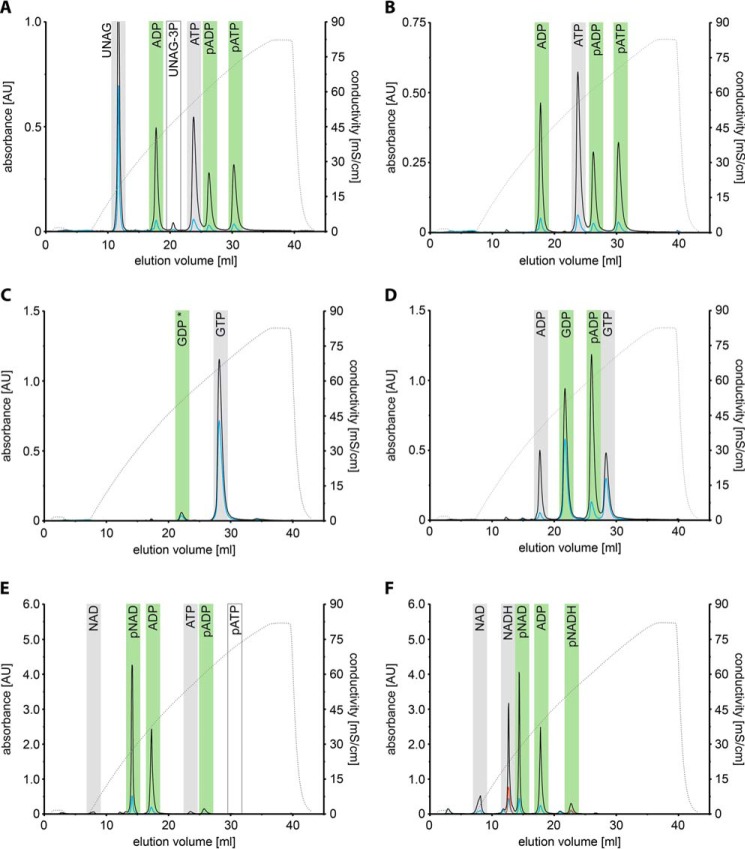
**AvrRxo1 phosphorylates adenine- but not guanine-containing nucleotides.** Different nucleotides (each at 500 μm) were tested as potential donor and acceptor substrates (*gray bars*) of AvrRxo1. After 5 h of incubation at 25 °C, products were detected by separation on an anion exchange chromatography column. Products of the reactions are marked with *green bars*. Traces shown are of *A*_260_ (*black*), *A*_280_ (*blue*), *A*_340_ (*red*), and eluate conductivity (*dotted gray line*). *A*, incubation of AvrRxo1 with UNAG and ATP does not lead to significant amounts of phosphorylated UNAG but results in accumulation of ADP and two apparently phosphorylated ADP and ATP species (*pADP* and *pATP*). *B*, similarly, incubation of AvrRxo1 with ATP alone results in accumulation of ADP and phosphorylated ADP and ATP species. *C*, when incubated with GTP alone, neither GDP nor any other nucleotide species is formed. The detected GDP species was identified as an impurity of the GTP stock and is thus marked with an *asterisk. D*, in contrast, incubation of AvrRxo1 with equimolar amounts of GTP and ADP causes accumulation of GDP and phosphorylated ADP. AvrRxo1 thus utilizes GTP as a phosphate donor but accepts neither GDP nor GTP as phosphate acceptors in contrast to ADP. *E*, when incubated with ATP and NAD, nearly all NAD becomes phosphorylated (*pNAD*) by AvrRxo1, whereas phosphorylation of ADP and ATP is virtually absent. *F*, chasing equal amounts of NAD and NADH as phosphate acceptor candidates reveals that AvrRxo1 favors NAD under the experimental conditions. Note that any minor traces of ATP used in reaction *F* would co-elute with phosphorylated NADH (*pNADH*) and are therefore not indicated. *AU*, absorbance units.

Apart from the structural homology of this central AvrRxo1 domain with polynucleotide kinases, the recently reported 3D structure of AvrRxo1 revealed a strong homology of AvrRxo1 with UDP-*N*-acetylglucosamine (UNAG) kinases of the ϵ-ζ toxin-antitoxin family (19). This raised the question whether AvrRxo1 might be an authentic UNAG kinase or not as the previously identified, characteristic UNAG binding motif of ζ kinases (25) cannot be identified in the effector's primary structure (Fig. 1). In fact, neither we nor others (19) could show that AvrRxo1 catalyzes the transfer of the γ-phosphate group of ATP onto UNAG to a significant extent because only minor traces of a potential UNAG-3P species were detected when a reaction mixture of AvrRxo1 incubated with UNAG and ATP for 5 h was analyzed using anion exchange chromatography (Fig. 2*a*).

In contrast, we observed a significant accumulation of ADP and two new, apparently phosphorylated species, indicating that AvrRxo1 is a nucleotide kinase with hitherto uncharacterized substrate specificity (Fig. 2*a*). Formation of these new species was not dependent on the presence of UNAG as incubation of AvrRxo1 with ATP alone resulted in the accumulation of products with identical retention times (Fig. 2*b*). Determination of their molecular masses suggested that AvrRxo1 catalyzed the phosphorylation of ATP (*m*/*z*_obs_ = 586.0) and ADP (*m*/*z*_obs_ = 506.0) in these long term incubation assays. This activity could be assigned to AvrRxo1 because neither of these products accumulated when the catalytically impaired AvrRxo1(D193N) variant was incubated with ATP (supplemental Fig. S1).

Because AvrRxo1 harbors the conventional P-loop motif (26), we wondered whether the enzyme also utilizes guanine nucleotides as substrates. In contrast to ATP, however, neither GDP nor any new, potentially phosphorylated GTP/GDP species accumulated when AvrRxo1 was incubated with GTP alone (Fig. 2*c*). We then scrutinized substrate binding site specificities by incubating AvrRxo1 with equimolar amounts of GTP and ADP and found that ADP was phosphorylated under the consumption of GTP (Fig. 2*d*). The phosphate donor site of AvrRxo1 thus binds ATP as well as GTP, whereas the phosphate acceptor binding site is selective for adenine-containing nucleotides rather than guanine nucleotides.

Because we observed significant amounts of residual ATP even after 5 h of incubation at 25 °C with ATP alone (Fig. 2*b*), we searched for better, adenine-containing substrates. We found that nicotinamide adenine dinucleotide (NAD) was quantitatively phosphorylated by AvrRxo1 after 5 h of incubation, whereas only minor traces of the phosphorylated ADP species were detected (Fig. 2*e*). NAD must consequently be the superior substrate when compared with ATP or ADP. We next tested whether the oxidation state of the nicotinamide moiety influences phosphorylation efficiency and incubated AvrRxo1 with equimolar amounts of NAD and NADH and reaction-limiting concentrations of ATP. Nearly all NAD was found to be phosphorylated by AvrRxo1, whereas only a minor fraction of NADH was phosphorylated (Fig. 2*f*). This apparent selection between NAD and NADH by the acceptor substrate binding site under the experimental conditions strongly argues for NAD as a substrate being phosphorylated *in vivo*.

##### AvrRxo1 Expression Leads to Accumulation of Phosphorylated NAD in E. coli

The observed preferential phosphorylation of NAD *in vitro* prompted the question whether NAD is also the preferred substrate of AvrRxo1 *in vivo*. We therefore prepared small metabolite extracts (SMEs) from *E. coli* cells expressing the AvrRxo1 protein and separated them using anion exchange chromatography.

**FIGURE 3.**
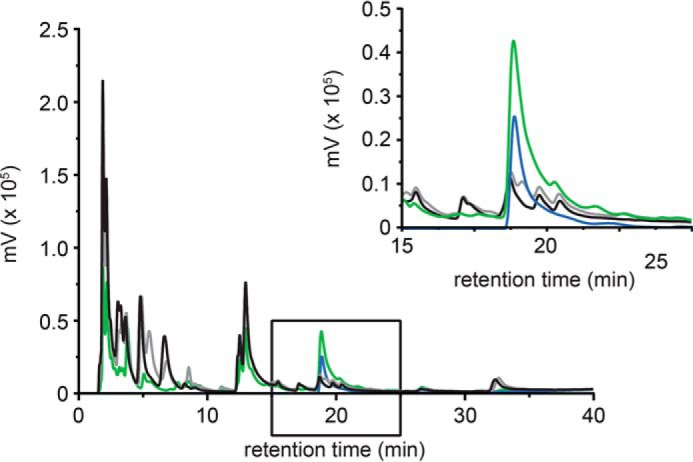
***E. coli* cells expressing AvrRxo1 accumulate a phosphorylated NAD species co-eluting with the enzymatic product obtained from *in vitro* reactions.** An anion exchange chromatogram shows *A*_260_ traces obtained by separation of small metabolite extracts prepared from *E. coli* cells. Traces shown are from extracts of untransformed cells (*black*), cells expressing AvrRxo1 (*green*), or cells expressing the catalytically impaired AvrRxo1(D193N) variant (*gray*). The phosphorylated NAD species (*blue*) prepared from *in vitro* reactions of AvrRxo1, ATP, and NAD was used as an authentic standard at 50 μm and co-elutes with the nucleotide species accumulating *in vivo* upon AvrRxo1 expression (*inset*). Note that at retention times 5.5 and 8 min two additional species accumulate in extracts from both the catalytically impaired AvrRxo1(D193N) and the wild type AvrRxo1 protein. Most likely, these are expression artifacts and not related to AvrRxo1 catalysis.

**FIGURE 4.**
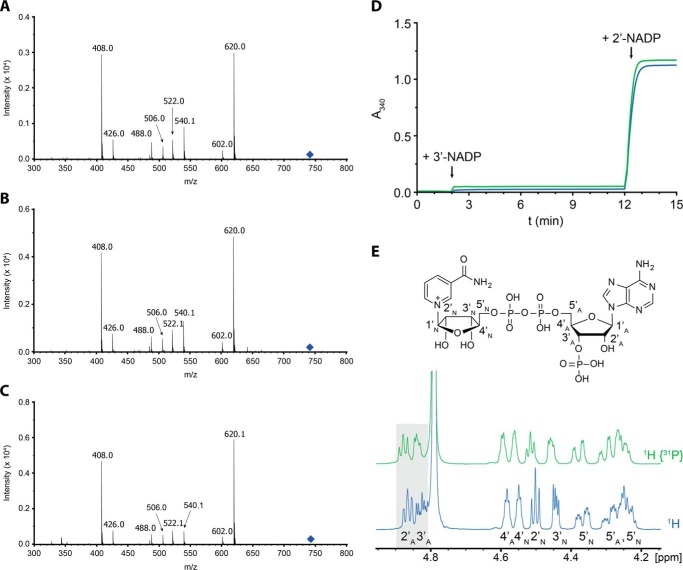
**AvrRxo1 catalyzes the formation of 3′-phosphorylated NAD.** Purified reaction products of AvrRxo1 *in vitro* reactions and small metabolite extracts were used for characterization by ESI MS-MS, a spectrophotometric assay using the 2′-NADP-dependent enzyme G6P-DH, and NMR spectroscopy. *A–C*, ESI MS-MS spectra were obtained by selecting the 742.1/743.1-Da heavy, phosphorylated NAD/NAAD species (*blue diamonds*) and fragmenting them at −45 and −40 eV, respectively. *A*, ESI MS-MS spectrum of phosphorylated NAD purified from *in vitro* reactions. The detected fragments correspond to those obtained by fragmenting phosphorylated NAD from SMEs (*B*) and *in vitro* phosphorylated NAAD (*C*). Fragments detected can be ascribed to phosphorylated ADP-ribose (620.0 Da), ADP-ribose (540.1 Da), ATP/phosphorylated ADP (506.0 Da), and ADP (408.0 Da). For a structural representation of the obtained fragments, see supplemental Fig. S3. *D*, using the 2′-NADP-dependent enzyme G6P-DH, the phosphorylated products of AvrRxo1 were shown to be differently phosphorylated than conventional 2′-NADP. Neither addition of 200 μm *in vitro* phosphorylated NAD (*blue*) nor addition of the *in vivo* product to 200 μm (*green*) could stimulate G6P-DH activity. In contrast, 200 μm 2′-NADP were rapidly reduced to 2′-NADPH by the enzyme as shown by the increase in absorbance at 340 nm. Hence, AvrRxo1 produces a compound different from 2′-NADP but with identical mass. *E*, NMR characterization of *in vitro* phosphorylated NAD showed that the phosphate group transferred by AvrRxo1 is situated at the 3′-hydroxyl group of adenosine (*3*′*_A_*). Although the spectrum for the 3′-proton signal of adenosine (*3*′*_A_*) showed increased peak multiplicity due to ^1^H-^31^P coupling in the ^1^H NMR experiment when compared with the ^1^H-^31^P decoupled spectra, the spectrum for the 2′-proton signal (*2*′*_A_*) remained unchanged in both experiments (5.0–4.8 ppm and highlighted in *gray*). The entire spectra are shown in supplemental Fig. S4.

We found that cells expressing active AvrRxo1 contained a significant amount of a single nucleotide species with similar retention time as the phosphorylated NAD standard produced *in vitro*. In contrast, no such species could be detected in SMEs prepared from untransformed or AvrRxo1(D193N)-expressing cells (Fig. 3). To further characterize this nucleotide species, we isolated it in a semipreparative manner from large scale SMEs. Electrospray ionization mass spectrometry revealed identical masses for the accumulated small metabolite and the *in vitro* produced, phosphorylated NAD (*m*/*z* = 742.1) (supplemental Fig. S2), and collision-induced dissociation gave comparable fragmentation patterns (Fig. 4, *a* and *b*). Additionally, the obtained fragments allowed assigning the site of phosphorylation to the hydroxyl groups of the adenosine moiety.

##### AvrRxo1 Catalyzes the Phosphorylation of the 3′-Hydroxyl Group of the Adenosine Moiety of NAD

Phosphorylation of NAD at the adenosine ribose moiety by AvrRxo1 could either lead to conventional 2′-NADP or a novel, small metabolite, 3′-NADP. We thus probed phosphorylated NAD purified from AvrRxo1-expressing *E. coli* cells and the *in vitro* counterpart for their capability to stimulate activity of the 2′-NADP-dependent enzyme d-glucose-6-phosphate dehydrogenase similarly as described before (27). To our surprise, neither of the two compounds could be reduced by the enzyme (Fig. 4*d*), thereby strongly suggesting that AvrRxo1 catalyzes the formation of 3′-NADP and not conventional 2′-NADP.

To directly prove that phosphorylation catalyzed by AvrRxo1 occurs at the 3′-hydroxyl group of the adenosine moiety, we isolated NAD phosphorylated by AvrRxo1 *in vitro* in a preparative setup and elucidated its structure by 1D and 2D NMR experiments. ^31^P experiments clearly confirmed the presence of a third phosphate group, but the recorded proton shifts differed significantly from those reported for 2′-NADP (28), implying that the 3′-hydroxyl group of the adenosine moiety is being phosphorylated by AvrRxo1 (Fig. 4*e*). Ultimately, ^1^H-^31^P decoupling experiments together with ^1^H-^1^H COSY (supplemental Fig. S4) confirmed that the phosphate group transferred onto the NAD molecule by AvrRxo1 is located at the 3′-hydroxyl group of the adenosine moiety.

##### ADP Production by AvrRxo1 Is Stimulated by NAD and NADH

**FIGURE 5.**
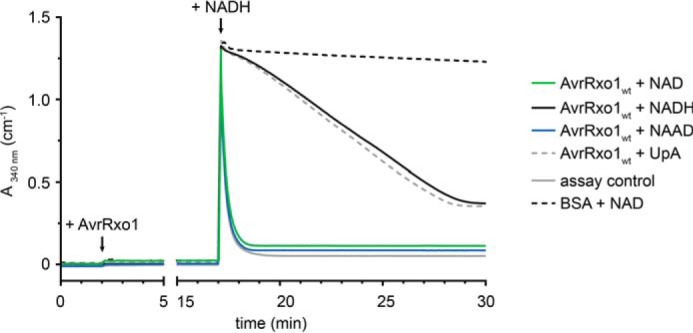
**ADP formation by AvrRxo1 is stimulated by NAD and NAAD but not by the structurally similar dinucleotide UpA.** A burst/chase experiment using the PK/LDH assay shows that conversion of ATP to ADP by AvrRxo1 is stimulated by NAD, NAAD, and NADH but not by the dinucleotide UpA. AvrRxo1 was added as indicated to a reaction mixture including PK, LDH, PEP, ATP, and a potential phosphate acceptor substrate or water. The reaction was incubated for 15 min to allow accumulation of pyruvate through ATP regeneration by PK from any newly formed ADP upon consumption of PEP. Subsequently, 200 μm NADH were titrated into the setup, and the absorbance at 340 nm was measured. Traces of *A*_340_ shown are of reactions in which the following compounds were present: H_2_O (*black*), NAD (*green*), NAAD (*blue*), UpA (*dotted gray*). The reaction setup with ATP, NAD, and no AvrRxo1 (*dotted black*) showed negligible activity. The maximum assay velocity was determined by incubation with 200 μm ADP (*gray*) for 15 min prior to NADH addition.

To compare the kinetics of ADP production arising from 3′-NADP formation with those of ATP phosphorylation, we scrutinized AvrRxo1 activity using a quantitative, spectroscopic ATPase steady state assay (29). Briefly, ATP was regenerated from newly formed ADP by pyruvate kinase (PK) upon conversion of phosphoenolpyruvate (PEP) to pyruvate. The latter was converted to lactate by lactate dehydrogenase (LDH) upon oxidation of NADH. During all experiments, PK, LDH, and PEP were adjusted to concentrations that allow the background reaction of the detection system to proceed much faster than AvrRxo1, which was checked by ADP control titrations (Fig. 5). Because we knew from previous experiments that NADH, ADP, and ATP can be phosphorylated by AvrRxo1, the assay was performed as a burst/chase experiment. To this end, AvrRxo1 was incubated with either ATP and NAD or ATP alone for 15 min. NADH was then titrated into the reaction setup at equimolar concentrations, and the decrease in absorbance at 340 nm was monitored.

Because we identified NAD as a suitable substrate, we expected that incubation with ATP and NAD would lead to production of ADP prior to NADH addition. Hence, pyruvate would accumulate substantially and LDH would become the rate-limiting factor, causing a drastic decrease in NADH absorbance (burst). In contrast, incubation with ATP alone should yield minor traces of ADP, and significant amounts thereof are only formed after NADH addition to the assay. Here, AvrRxo1 becomes rate-limiting over LDH, and the decrease in absorbance is much slower.

As expected, this experimental setup also revealed that NAD is preferred over ATP (Fig. 5). Because AvrRxo1 was recently suggested to be a polynucleotide kinase based on structural homology (19), we used this assay to compare a short polynucleotide analog and NAD as potential substrates. To structurally be as homologous as possible to NAD, we tested uridine 5′ → 3′ adenine dinucleotide (UpA) but found that incubation with ATP and UpA did not lead to production of any detectable amounts of ADP prior to NADH addition (Fig. 5). Hence, polynucleotides are unlikely the authentic substrates of AvrRxo1.

##### AvrRxo1 Phosphorylates NAD and NAAD with Similar Efficiency

**FIGURE 6.**
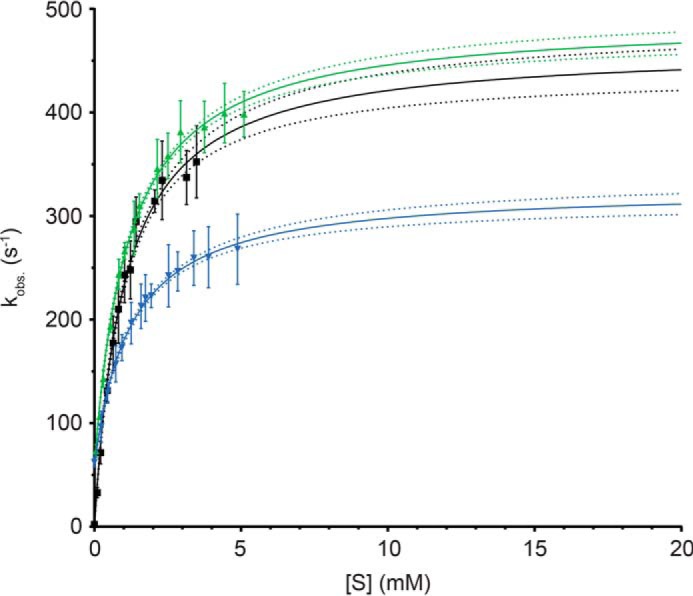
**AvrRxo1 is a highly efficient NAD/NAAD kinase.** Steady state Michaelis-Menten kinetics using the PK/LDH assay revealed that the *K_m_* values of AvrRxo1 for NAD (*green*) and NAAD (*blue*) are identical at 1.2 ± 0.1 mm at saturating Mg^2+^-ATP concentrations (3 mm). Turnover by the enzyme is rapid with *k*_cat_ values of 430 ± 10 s^−1^ for NAD and 270 ± 10 s^−1^ for NAAD. A comparable *k*_cat_ for ATP (*black*) at saturating NAD concentrations (4 mm) of 460 ± 10 s^−1^ was determined. The *K_m_* for ATP is slightly lower as for NAD with a value of 1.0 ± 0.1 mm. S.E. of triplicates are given as *bars* for each point measured. The 95% confidence interval for each fit is indicated by *dotted lines*.

Having confirmed that AvrRxo1 is a novel type of kinase that phosphorylates NAD *in vitro* and *in vivo*, we wondered whether it also accepts the biochemical precursor of NAD, NAAD, as an acceptor substrate. We thus tested NAAD as described in the previous section and found that it stimulates ADP production by AvrRxo1 similarly to NAD (Fig. 5). We therefore performed steady state ATP hydrolysis assays to characterize AvrRxo1 with regard to its kinetic parameters *K_m_* and *k*_cat_ for ATP, NAD, and NAAD (Fig. 6 and Table 1).

**TABLE 1:** **Kinetic properties of AvrRxo1** The values are derived from the fit of the arithmetic mean of independent triplicates, and their respective error within the 95% confidence interval is given. Kinetic parameters of ATP were determined at 4 mm NAD, and those of NAD and NAAD were determined at 3 mm Mg^2+^-ATP. The individual substrates for which the kinetic parameters were determined are given in bold characters, whereas the subscript indicates the substrate that was added at saturating conditions. App., apparent.

Substrate	App. *k*_cat_	App. *K_m_*	*k*_cat_/*K_m_*
	*s*^−*1*^	*mm*	
ATP_NAD_	460 ± 10	1.0 ± 0.1	460
NAD_ATP_	430 ± 10	1.2 ± 0.1	358
ATP_NAAD_*^a^*	340 ± 20	1.0 ± 0.1	340
NAAD_ATP_	270 ± 10	1.2 ± 0.1	225

*^a^* These values were derived from single measurements.

For the phosphate donor ATP, we determined the *K_m_* under saturating NAD concentrations to be 1.0 ± 0.1 mm, whereas the apparent turnover rate (*k*_obs_) is 460 ± 10 s^−1^. Strikingly, the *K_m_* for NAD and NAAD at saturating ATP concentration was determined to be identical at 1.2 ± 0.1 mm, and the *k*_cat_ values were 430 ± 10 s^−1^ for NAD and 270 ± 10 s^−1^ for NAAD. Hence, the specificity constants of AvrRxo1 for NAD and NAAD are comparable with values of 358 and 225 s^−1^ mm^−1^, respectively. AvrRxo1 therefore phosphorylates NAD and NAAD with similar efficiency. Which of the two compounds is phosphorylated *in vivo* consequently depends on the respective subcellular concentrations of these dinucleotides and the localization of the effector.

Furthermore, this reveals that AvrRxo1 is a highly potent NAD/NAAD kinase, especially when compared with the NAD kinase from *E. coli* that catalyzes the phosphorylation of NAD to 2′-NADP with a *k*_cat_ of 125 s^−1^ and a *K_m_* of 2 mm (*k*_cat_/*K_m_* = 62.5 s^−1^ mm^−1^) (30). It is important to note that the NAD concentrations used in NAD titration assays had to be corrected by a constant amount of NAD produced by the PK/LDH system due to an ADP impurity of the ATP stock (1.9%). The increase in NAD and concomitant decrease in NADH concentrations were identical for all NAD titration experiments and were determined to be 56 μm. Intriguingly, the PK/LDH system ensured constant NAD and ATP concentrations throughout each measurement, *i.e.* as long as NADH was present, representing ideal steady state conditions. In addition, NADH contributed to the observed initial velocities of each measurement although it is phosphorylated to a much lesser extent than NAD (Fig. 2*f*). In a first approximation, we assumed this to be constant in all measurements and therefore included an additive, basal background reaction, *k*_basal_, in the Michaelis-Menten equation (see “Experimental Procedures”). This basal background activity causes the fit not to intersect with the origin for NAD and NAAD titrations as would be the case for an unmodified Menten curve (Fig. 6).

##### AvrRxo2 Is a Potent, Mixed Inhibitor of AvrRxo1

Having revealed that AvrRxo1 is a potent 3′-NAD/NAAD kinase, we decided to investigate the effect of the second polypeptide encoded by the *AvrRxo1*/*2* bicistron, AvrRxo2, on the kinase activity of AvrRxo1. Co-expression with small polypeptides from a bicistron is a common hallmark of bacterial type III effectors (31). The majority of these small proteins interact with a single, cognate effector and are involved in prevention of detrimental interactions between the effector and other proteins, effector stabilization, and regulation of effector secretion (31). Similarly, AvrRxo2 has been proposed to act as such a chaperone-like protein (20). Recent findings, however, are somewhat contradictory to a pure chaperone function of AvrRxo2 in that co-expression of AvrRxo2 and AvrRxo1 suppressed the bacteriostatic phenotype observed for *E. coli* cultures expressing only AvrRxo1 (19).

**FIGURE 7.**
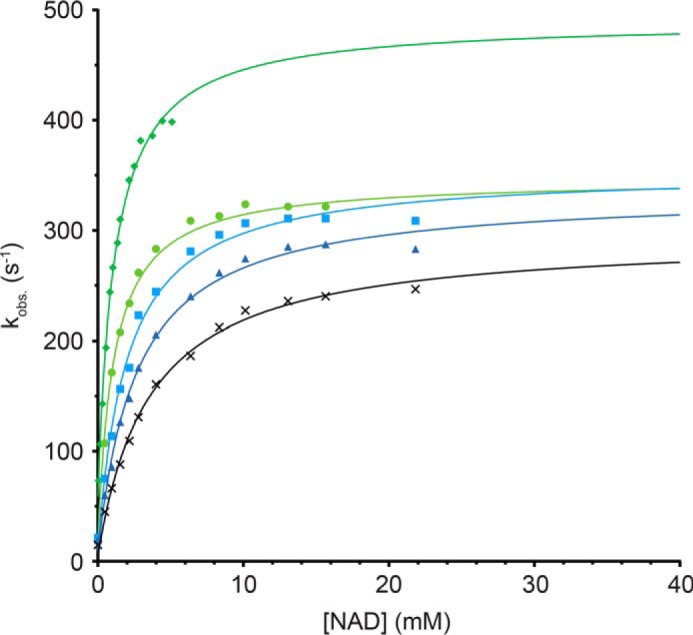
**AvrRxo2 is a potent inhibitor of AvrRxo1.** A reaction setup similar to that described for Fig. 6 was used to determine the effect of AvrRxo2 on AvrRxo1 kinase activity. A constant 2.5 nm AvrRxo1 together with 3 mm Mg^2+^-ATP was incubated with different concentrations of AvrRxo2: 0 (*dark green diamonds*), 2.5 (*light green circles*), 5.0 (*light blue squares*), 7.5 (*dark blue triangles*), and 12.5 nm (*black crosses*). NAD was titrated into the reactions, and the initial velocities of each reaction were determined and plotted. Equimolar concentrations of AvrRxo2 already have a dramatic effect on AvrRxo1 kinase activity, revealing AvrRxo2 as a potent inhibitor of the effector. Because both *k*_cat_ and *K_m_* of AvrRxo1 are affected, AvrRxo2 acts as a mixed inhibitor.

Using the spectroscopic ATPase assay, we found that the apparent *k*_cat_ of AvrRxo1 for NAD was already strongly affected at equimolar concentrations of AvrRxo1 and AvrRxo2 (Fig. 7). Furthermore, also the *K_m_* was found to increase proportionally with AvrRxo2 concentration, showing that AvrRxo2 interferes as a mixed inhibitor with AvrRxo1 kinase activity. Both proteins might therefore constitute an effector/immunity pair (32) in *Xanthomonas*.

## Discussion

The bacterial type III effector protein AvrRxo1 has recently been identified as an important virulence factor of the rice pathogen *X. oryzae* pv. *oryzicola* (20). Strikingly, it is also found in the genome of other pathogenic bacteria that infect crops from different plant families (Fig. 1). Despite recent insights into the role of AvrRxo1 in plant-pathogen interactions (20, 21), its enzymatic properties remained enigmatic so far. The recent structure-based speculation that AvrRxo1 might be a polynucleotide kinase (19) disagrees with our finding that no ADP formation by AvrRxo1 could be induced even in the presence of the short dinucleotide UpA. Instead, we showed that AvrRxo1 is a novel type of kinase that modifies the coenzyme NAD and its biochemical precursor NAAD (Figs. 4 and 5), whereas NADH is phosphorylated with much lower efficiency (Fig. 2*f*).

We verified that phosphorylation occurs at the adenosine 3′-hydroxyl group of NAD by NMR characterization of the enzymatic product (Fig. 4*e*). AvrRxo1 thus catalyzes the formation of two previously unknown natural compounds, 3′-NADP and 3′-NAADP, that are different from conventional 2′-NADP. We additionally provide strong evidence that AvrRxo1 also produces significant amounts of 3′-NADP *in vivo*.

The first reported chemical synthesis of 3′-NADP dates back to the mid-1950s when it was produced by acidic isomerization of 2′-NADP (27). Since then, 3′-NADP was tested as a potential synthetic inhibitor for a variety of NAD(P)-dependent enzymes (27, 33–35). In the last two decades, however, 3′-NADP was orphaned, probably due to its surmised artificial character. We now show that AvrRxo1 effectors secreted by plant pathogens produce high levels of this compound in living organisms, and thus 3′-NADP needs to be included in the league of natural compounds. Our studies open the possibility for further elaborated *in vivo* studies by enzymatically producing this compound upon AvrRxo1 expression.

Interestingly, 3′-NADP was shown to be a potent, non-competitive inhibitor of the maize NADP-dependent malic enzyme with a *K*_is_ of 56.7 μm (36). NADP-dependent malic enzymes play a central role in the carbon fixation process of globally important C_4_ plants by catalyzing the decarboxylation of malate to pyruvate and CO_2_ in bundle sheath cells (37). It is tempting to speculate that, after being injected into the host cell, AvrRxo1 might interfere with carbon fixation pathways in C_4_ plants and thereby establishes cytotoxicity. Consequently, certain cultivars of the C_4_ plant maize might have evolved an Rxo1 resistance protein that establishes ETI when exposed to AvrRxo1 (38). The finding that the maize pathogen *P. andropogonis* encodes an AvrRxo1 homolog suggests that this ETI program has evolved against infections with *P. andropogonis* rather than *X. oryzae* pv. *oryzicola*.

In contrast, the C_3_ plant rice suffers from leaf streak and apparently does not elicit an ETI once infected with *X. oryzae* pv. *oryzicola*, although it encodes an Rxo1 protein highly similar to that of maize (39). This might reflect that the selective pressure on C_3_ plants by inhibition of NADP malic enzymes is lower than on C_4_ plants.

Furthermore, our kinetic studies showed that the second potential product of AvrRxo1 catalysis is 3′-NAADP because phosphorylation of NAD and NAAD has similar *in vitro* kinetic properties. NAAD is a low abundance metabolite that is converted to NAD by NAD synthetase. This occurs in *E. coli* with high efficiency, causing extremely low cellular levels of NAAD when compared with NAD concentrations (40). Thus, we did not expect to detect significant amounts of 3′-NAADP in our SME analysis. Similar to 3′-NADP, 3′-NAADP is known since the mid-1970s as a synthetic compound produced by acidic isomerization of 2′-NAADP (41). The latter is an established and highly potent Ca^2+^-mobilizing second messenger in animals and plants (42–44). Interestingly, 3′-NAADP also is highly potent in mobilizing Ca^2+^ ions (45, 46). Similar to 3′-NADP, 3′-NAADP was orphaned due to its synthetic character and has not been further investigated as a potential Ca^2+^-mobilizing second messenger. Based on our findings, however, this might need some reconsideration.

Nevertheless, to what extent 3′-NAADP is formed after translocation of AvrRxo1 into the plant cell cytosol needs to be determined as the cellular NAAD concentrations are low. However, even small amounts of 3′-NAADP should suffice to interfere with the intracellular “Ca^2+^ signature” because the half-maximal effective concentration of 3′-NAADP for triggering a Ca^2+^ release in sea urchin eggs was shown to be approximately 300 nm (46). The finding that AvrRxo1 catalyzes the formation of the Ca^2+^ releaser 3′-NAADP is particularly intriguing because Ca^2+^ signaling is a highly sensitive, universal signal transduction mechanism in eukaryotes (47, 48), and Ca^2+^ influx from the apoplast is a characteristic process in plant cells initiating an HR against invading pathogens (49, 50).

It thus seems plausible that 3′-NAADP produced by AvrRxo1 interferes with host cell immune responses by manipulating the intracellular Ca^2+^ signature. Further substantiating this hypothesis, the non-host HR of tobacco against *X. oryzae* pv. *oryzae* could be suppressed by transforming the bacteria with an AvrRxo1-encoding plasmid (21). A curious side note is that structural studies on the type III effector XopQ from *X. oryzae* pv. *oryzae* suggested a nucleoside hydrolase activity on the Ca^2+^-mobilizing second messenger cyclic adenosine diphosphate ribose (51). Hence, manipulation of the host cell Ca^2+^ signature might not only be performed by AvrRxo1 but could be a common approach of plant pathogens.

We have exemplarily discussed potential scenarios how 3′-NADP and 3′-NAADP production by AvrRxo1 could enhance virulence of plant pathogens. However, effector molecules do not necessarily promote pathogenicity by targeting a single cellular pathway but frequently do so by simultaneously interfering with multiple cellular processes (1). Both products of AvrRxo1 might thus also interfere with a vast array of other, pivotal biological processes requiring NAD and its derivatives as co-enzymes. Such processes are for instance anabolic and catabolic pathways as well as regulation of gene expression by posttranslational modifications of proteins including mono/poly(ADP-ribosyl)ation (52) and histone deacetylation (53). Interestingly, NAD is also required for the synthesis of cyclic ADP-ribose, another highly potent Ca^2+^ releaser, by ADP-ribosylcyclases (54).

Revealing the enzymatic properties of AvrRxo1 enabled us to further characterize the function of AvrRxo2. It has previously been shown that co-expression of AvrRxo2 and AvrRxo1 cures the bacteriostatic phenotype observed when cells express AvrRxo1 alone (19). We showed that AvrRxo2 strongly inhibits AvrRxo1 nucleotide kinase activity, thereby most likely protecting the pathogen from cytotoxic effects due to AvrRxo1 expression, similar to common effector/immunity systems. We therefore assume that AvrRxo1 becomes liberated from AvrRxo2 just before translocation via the type III secretion system and consequently exerts its detrimental effects exclusively within the plant host cell.

In conclusion, we provide experimental evidence that AvrRxo1 is a novel type of kinase that phosphorylates NAD and NAAD at the 3′-hydroxyl groups of their adenosines. Both products of the effector, 3′-NADP and 3′-NAADP, which were so far assumed to be purely synthetic, are thus also found as natural compounds produced by plant pathogens. As bacterial effector proteins like AvrRxo1 have evolved to subversively manipulate critical host cell processes, the identification of their mechanism provides insights into conserved eukaryotic pathways and greatly contributes to advances in the field investigating host-pathogen interactions.

## Experimental Procedures

### 

#### 

##### Cloning, Expression, and Purification of AvrRxo1 and AvrRxo2 Constructs

The construct pGEX4T-1_AvrRxo1ΔN88 encoding a truncated AvrRxo1 variant (henceforth referred to as AvrRxo1) from *X. oryzae* pv. *oryzicola* was generated by introduction of a BamHI restriction site into pGEX4T-1_AvrRxo1(ΔN65) (19) (kindly provided by B. Zhao, Virginia Tech) before the Thr-89 codon following the QuikChange protocol (Agilent Technologies, Santa Barbara, CA) with primer pair Avr1Bam_f/r (see Table 2 for all primers). The coding region for residues 66–88 was removed by religating the BamHI-digested plasmid. The BamHI restriction site was then removed by site-directed mutagenesis using primer pair Avr1BamX_f/r. The plasmid pGEX4T-1_AvrRxo1ΔN88 (D193N) encoding the catalytically impaired AvrRxo1 variant AvrRxo1 (D193N) was obtained by site-directed mutagenesis of pGEX4T-1_AvrRxo1ΔN88 using primer pair Avr1QC_D193N_f/r.

**TABLE 2:** **Primers used for cloning of AvrRxo1 and AvrRxo2 constructs**

Primer	Sequence
Avr1Bam_f	CGCGGTGACACATCAGGATCCTACTCAGCGTAAAGG
Avr1Bam_r	CCTTTACGCTGAGTAGGATCCTGATGTGTCACCGCG
Avr1BamX_f	CCCCTTCACCGGAACTCAGCGTAAAGGC
Avr1BamX_r	GCCTTTACGCTGAGTTCCGGTGAAGGGG
Avr1QC_D193N_f	GGTTGCGTGAATCCAAATGTATTCAAGAGTTCG
Avr1QC_D193N_r	CGAACTCTTGAATACATTTGGATTCACGCAACC
Avr2_NdeI	GCAACATATGAAAACTTTGACAGG
M13_rev	CAGGAAACAGCTATGAC
Avr2QC_W97A_f	CGAGACACTTTTCTCGGCGTCAGTCGACCAC
Avr2QC_W97A_r	GTGGTCGACTGACGCCGAGAAAAGTGTCTCG
Avr2_dN25_NdeI	GATTCGAGCATATGGACCTCGAGTTAGCACTGGTG
Avr2_STOP_rev	AAGTCAGTGCGGCCGCTCATGACCACGAGAAAAGTGTCTCG

Two different translational start sites have been predicted for the ORF of AvrRxo2 from *X. oryzae* pv. *oryzicola*, giving two hypothetical AvroRxo2 polypeptide chains with either 117 amino acids (AA) (NCBI entry AEQ98134) or 98 AA (NCBI entry WP_041183485) in length. We purchased plasmid pMA(AEQ98134) containing a synthetic gene for the ORF of the 117-AA AvrRxo2 variant flanked at the 5′-end with a start codon overlapping the NcoI restriction site, a 3′-terminal coding sequence for a hexahistidine tag just before the stop codon, and a BamHI restriction site downstream thereof (Life Technologies^TM^, Thermo Fisher Scientific, Waltham, MA). The synthetic gene was excised by a restriction digest with NcoI and BamHI and ligated into pET28b (pET28b_AEQ98134). Expression and purification using pET28b_AEQ98134 revealed that both AvrRxo2 variants are equally expressed from this construct, indicating that a functional Shine-Dalgarno sequence and a start codon for the 98-AA AvrRxo2 variant are present (data not shown). We thus decided to exclusively use the 98 AA-encoding ORF (WP_041183485) in all our experiments and will refer to its product as AvrRxo2 similarly as described before (19). To this end, DNA for the AvrRxo2 coding sequence was PCR-amplified from pMA(AEQ98134) using primers Avr2_NdeI and M13_rev. Following an NdeI and BamHI restriction digest, the fragment was ligated into a pET24b vector (pET24b_AvrRxo2). This plasmid was used to generate pET24b_AvrRxo2(W97A) by site-directed mutagenesis using primer pair Avr2QC_W97A_f/r. The expression construct pET24b_AvrRxo2ΔN25 encoding an AvrRxo2 variant truncated by the first 25 residues and lacking the C-terminal His tag was obtained by PCR amplification of the encoding region from pET24b_AvrRxo2 plasmid DNA using primers Avr2_dN25_NdeI and Avr2_STOP_rev. Subsequently to a restriction digest with NdeI and NotI, the PCR product was ligated into an equivalently cut pET24b expression vector. The identity and correctness of all expression vectors were verified by sequencing of purified plasmid DNA.

For expression and purification of AvrRxo1 and AvrRxo2 constructs, *E. coli* BL21(DE3)-RIL cells were either transformed with vector pET24b_AvrRxo2 or pGEX4T-1_AvrRxo1ΔN88(D193N) alone or with vector pGEX4T-1_AvrRxo1(ΔN88) together with either pET24b_AvrRxo2(ΔN25) or pET24b_AvrRxo2(W97A). Cells were grown at 37 °C overnight on Luria-Broth (LB) medium agar plates containing 0.1 mg/ml ampicillin, 0.05 mg/ml kanamycin, or both in the case of co-expression. A liquid preculture of LB medium containing 0.1 mg/ml ampicillin and/or 0.05 mg/ml kanamycin was grown to exponential phase at 37 °C and used as inoculum for expression cultures. Final cell cultures for expression were grown to an *A*_600_ of 0.3–0.4 at 37 °C, cooled to 16 °C, and further incubated until reaching an *A*_600_ of 0.6. Protein expression was induced by addition of 0.5 mm isopropyl β-d-1-thiogalactopyranoside. Cells were harvested by centrifugation after overnight expression, and pellets of all expression constructs were suspended in cold buffer A (50 mm Tris-HCl, pH 8.5, 150 mm NaCl, 50 mm (NH_4_)_2_SO_4_, 5 mm β-mercaptoethanol (β-me)). All subsequent purification steps were performed at 4 °C or on ice. Cell walls were broken by sonication, and the lysate was cleared by centrifugation. The supernatant was further filtered through a 0.45-μm-cutoff syringe filter before being loaded onto the first column.

Because AvrRxo1 proved to be poorly soluble when expressed alone in *E. coli*, we co-expressed AvrRxo1 together with the N-terminally truncated AvrRxo2ΔN25 variant. This substantially improved AvrRxo1 solubility during expression and allowed removal of AvrRxo2ΔN25 due to its reduced affinity for AvrRxo1 compared with full-length AvrRxo2. To purify AvrRxo1, the filtered supernatant was loaded onto a 1-ml-column volume (CV) HisTrap Ni-NTA column (GE Healthcare) equilibrated with buffer A. The column was washed with 10 CVs of buffer B (50 mm Tris-HCl, pH 8.5, 150 mm NaCl, 50 mm (NH_4_)_2_SO_4_, 30 mm imidazole, 2 mm dithioerythritol (DTE)). The majority of AvrRxo2ΔN25 was in the flow-through. Bound protein was eluted in 5 CVs of buffer C (50 mm Tris-HCl, pH 8.5, 150 mm NaCl, 50 mm (NH_4_)_2_SO_4_, 200 mm imidazole, 2 mm DTE). The eluate was supplemented with 30 μg/ml TEV protease to remove the N-terminal GST and the C-terminal hexahistidine tags of AvrRxo1. The eluate was then dialyzed overnight against buffer D (50 mm Tris-HCl, pH 8.5, 100 mm NaCl, 10 mm (NH_4_)_2_SO_4_, 2 mm DTE). Precipitated protein was removed by centrifugation, and the supernatant was applied onto a 0.5-ml-CV Ni-NTA-agarose (Qiagen, Hilden, Germany) gravity flow column equilibrated with buffer D to remove the hexahistidine-tagged TEV protease and residual, still tagged AvrRxo1 protein. The flow-through was diluted to a conductivity of 10–11 mS/cm with buffer E (50 mm Tris-HCl, pH 8.5, 2 mm DTE) and loaded onto a 1-ml-CV HiTrap Heparin HP column (GE Healthcare) equilibrated with buffer E. Protein was eluted with a linear gradient of 20 CVs to buffer F (50 mm Tris-HCl, pH 8.5, 400 mm NaCl, 2 mm DTE). Pure protein fractions were pooled, concentrated in a 10,000-molecular weight-cutoff centrifugal filter and loaded onto a Superdex 75 10/300 GL column (GE Healthcare) equilibrated with buffer G (50 mm HEPES-NaOH, pH 7.5, 200 mm NaCl, 2 mm DTE) for final polishing and buffer exchange. The catalytically impaired, non-toxic AvrRxo1(D193N) variant could be expressed alone and was purified following the same protocol.

Similarly to AvrRxo1, the cleared supernatant from AvrRxo2 expression was loaded onto a 1-ml-CV Ni-NTA-agarose column (Qiagen) gravity flow column equilibrated with buffer A. A wash step of 10 CVs of buffer H (50 mm Tris-HCl, pH 8.5, 150 mm NaCl, 50 mm (NH_4_)_2_SO_4_, 5 mm imidazole, 5 mm β-me) was performed. Bound protein was eluted in 7 CVs of buffer C. To improve the yield, the flow-through of the first Ni-NTA purification step was rechromatographed. Both eluates were pooled and, the conductivity was adjusted to 10–11 mS/cm with buffer I (50 mm Tris-HCl, pH 8.0, 20 mm (NH_4_)_2_SO_4_, 2 mm DTE). To remove anionic species and contaminating oligonucleotides, AvrRxo2 was passed through a Mono Q 10/100 GL column (GE Healthcare) equilibrated with buffer I. The flow-through was concentrated by binding to a 1-ml-CV HisTrap Ni-NTA column (GE Healthcare) equilibrated with buffer A and eluted from the column with buffer J (50 mm Tris-HCl, pH 8.5, 150 mm NaCl, 50 mm (NH_4_)_2_SO_4_, 250 mm imidazole, 2 mm DTE). The AvrRxo2-containing eluate was dialyzed overnight against buffer K (50 mm Tris-HCl, pH 8.0, 150 mm NaCl, 50 mm (NH_4_)_2_SO_4_, 5 mm DTE). Finally, the protein was concentrated in a 3,000-molecular weight-cutoff centrifugal filter and loaded onto a Superdex 75 10/300 GL column (GE Healthcare) equilibrated with buffer G.

The cleared supernatant of AvrRxo1/AvrRxo2(W97A) co-expression was loaded onto a 0.8-ml-CV Ni-NTA-agarose (Qiagen) gravity flow column equilibrated with buffer A at pH 8.0. The column was washed with 12 CVs of buffer A at pH 8.0 supplemented with 2 mm DTE instead of β-me, and bound proteins were subsequently eluted with 6 CVs of buffer C at pH 8.0. The eluate was dialyzed overnight against buffer A at pH 8.0 and applied onto a 0.5-ml-CV glutathione-Sepharose 4 FF (GE Healthcare) gravity flow column equilibrated with buffer A at pH 8.0. A wash with 10 CVs of buffer L (50 mm Tris-HCl, pH 8.0, 150 mm NaCl, 50 mm (NH_4_)_2_SO_4_, 0.5 mm EDTA, 0.5 mm MgCl_2_, 2 mm DTE) was performed. Subsequently, the column material was suspended in 8 ml of buffer L supplemented with 1 mg of TEV protease and incubated at room temperature for 2.5 h under constant, mild agitation. The supernatant containing the complex of untagged AvrRxo1 and AvrRxo2(W97A) was collected, concentrated in a 10,000-molecular weight-cutoff centrifugal filter, and loaded onto a Superose 12 10/300 GL column (GE Healthcare) equilibrated with buffer G.

All purification procedures were monitored by Coomassie-stained 20% (w/v) SDS-PAGE, and purity of final protein batches was judged to be better than 98%. Protein concentrations of batches were determined on a Nanodrop (Thermo Fisher Scientific) using the following extinction coefficients at 280 nm: ϵ(AvrRxo1/AvrRxo1(D193N)) = 32,890 m^−1^ cm^−1^, ϵ(AvrRxo2) = 11,460 m^−1^ cm^−1^, and ϵ(AvrRxo2(W97A)) = 5,960 m^−1^ cm^−1^.

##### Phosphotransferase Assays

AvrRxo1 at 120 nm was probed for phosphotransferase activity in 200 μl of buffer M (50 mm HEPES-NaOH, pH 7.5, 200 mm NaCl, 15 mm KCl, 3 mm MgCl_2_, 1 mm EDTA, 1 mm TCEP supplemented with different nucleotides at 500 μm (ADP (Pharma Waldhof GmbH, Düsseldorf, Germany), GDP, UNAG (both from Sigma-Aldrich), UpA (IBA GmbH, Göttingen, Germany), NAD or NADH (both from Roche Diagnostics GmbH)), and equimolar amounts of ATP (Pharma Waldhof GmbH) or GTP (Sigma-Aldrich). Reactions were incubated for 5 h under mild agitation at 25 °C, quenched by freezing in liquid nitrogen, and stored at −80 °C. For analysis, samples were diluted with 2 ml of deionized water. The total sample was loaded onto a Mono Q 5/50 GL column (GE Healthcare) equilibrated with deionized water. Bound compounds were eluted in a linear gradient of 30 CVs to an aqueous solution of 1.5 m NH_4_^+^CH_3_COO^−^ at pH 8.0. Eluting species were detected by measuring absorbance at 260, 280, and 340 nm.

##### Detection of AvrRxo1 Products in Small Metabolite Extracts

Precultures of 50 ml of LB medium supplemented with 0.1 mg/ml ampicillin were inoculated with *E. coli* BL21(DE3)-RIL cells transformed with either pGEX4T-1_AvrRxo1ΔN88 or pGEX4T-1_AvrRxo1ΔN88(D193N) as a negative control and incubated at 37 °C. Cultures of untransformed cells were performed identically in LB medium without ampicillin. From these, final 500-ml expression cultures were inoculated and incubated at 37 °C until the *A*_600_ reached 0.2. The temperature was set to 16 °C, and protein expression was induced at an *A*_600_ of 0.3 by adding 0.5 mm isopropyl β-d-1-thiogalactopyranoside. Cells were harvested 90 min postinduction by centrifugation at 4 °C. Small metabolite extraction and HPLC analysis were performed as described previously (55).

##### In Vitro Preparation and Purification of AvrRxo1 Products

Co-purified AvrRxo1-AvrRxo2(W97A) complex at 21 nm was incubated in a preparative enzymatic setup together with 6.8 mm NAD and 7 mm Mg^2+^-ATP in 10 ml of buffer N (50 mm HEPES-NaOH, pH 7.5, 200 mm NaCl, 15 mm KCl, 7 mm phosphoenolpyruvate, 1 mm EDTA). An additional 1.4 units/ml PK and 2 units/ml LDH (Sigma-Aldrich) were added. The reaction was incubated at room temperature for 19 h in the dark. Proteins were removed by filtration through a 10,000-molecular weight-cutoff centrifugal filter. The filtrate was diluted with deionized water to a conductivity of 13 mS/cm, and 40-ml aliquots were loaded onto a Mono Q 10/100 GL column equilibrated with deionized water. Phosphorylated NAD was separated from ATP by a step elution with 400 mm ammonium acetate buffer at pH 8.0. For polishing, the eluate was diluted with the same volume of deionized water and reloaded onto the Mono Q 10/100 GL column equilibrated with water. Most impurities were removed in a first step gradient to 240 mm ammonium acetate buffer at pH 8.0. The phosphorylated NAD product was then eluted from the column with 350 mm ammonium acetate buffer at pH 8.0. Subsequently, the buffer was exchanged to the more volatile ammonium carbonate buffer at pH 8.0 by reloading phosphorylated NAD diluted in deionized water onto the Mono Q 10/100 GL column followed by a step elution with 150 mm ammonium carbonate buffer at pH 8.0. The purified product was concentrated under vacuum, and a brownish contaminant could be removed by 70% (v/v) ethanol precipitation, whereas the phosphorylated NAD remained in solution. Last traces of contaminants were removed by reloading the highly concentrated product onto a Mono Q 5/50 GL column and elution with 85 mm ammonium carbonate buffer at pH 8.0. After freeze-drying, the pure product was obtained as a colorless solid phase and dissolved in 600 μl of D_2_O for NMR characterization.

Phosphorylated NAAD was purified from reactions of Michaelis-Menten kinetic experiments. After measurements were taken, all reactions were pooled, frozen in liquid nitrogen, and stored at −80 °C. Purification of phosphorylated NAAD followed the same protocol as was used for purification of phosphorylated NAD with the only difference being that 600 mm NH_4_HCO_3_ at pH 8.0 was used for initial elutions.

##### Purification of 3′-NADP from Small Metabolite Extracts

Purification of 3′-NADP from SMEs was performed similarly as described for the *in vitro* product. Extracts were obtained from 3.5 liters of AvrRxo1-expressing *E. coli* culture identically as described above. 3′-NADP was purified by suspending the dried extracts in 10 ml of H_2_O and loading it onto a Mono Q 10/100 GL column equilibrated with H_2_O. The flow-through containing species less negatively charged than 3′-NADP was discarded, and bound species were eluted with a linear gradient of 0–1 m ammonium acetate buffer at pH 8.0 over 15 CVs. Eluate fractions were pooled and applied onto a Mono Q 5/50 GL column equilibrated with H_2_O. 3′-NADP was separated from other bound species by washing the column with 5 CVs of 300 mm ammonium acetate buffer at pH 8.0 and then eluting 3′-NADP with a linear gradient over 4 CVs of the buffer from 300 to 500 mm. Fractions containing 3′-NADP were again loaded onto a Mono Q 10/100 GL column equilibrated with H_2_O for buffer exchange to the more volatile ammonium carbonate. 3′-NADP was eluted with 350 mm ammonium carbonate buffer at pH 8.0. The obtained fractions were lyophilized and suspended in H_2_O until the sample no longer showed any traces of ammonia.

##### Electrospray Ionization Mass Spectrometry of AvrRxo1 Products

Molecular masses of AvrRxo1products purified from *in vitro* reactions and small metabolite extracts were determined on a Bruker maXis II mass spectrometer (Bruker Corp., Billerica, MA) following dilution in deionized water. Compounds were fragmented by collision-induced dissociation at −45 eV for phosphorylated NAD and −40 eV for phosphorylated NAAD.

##### d-Glucose-6-phosphate Dehydrogenase Assays of 3′-NADP

3′-NADP purified from *in vitro* reactions or small metabolite extracts was assayed for its capability to induce activity of d-glucose-6-phosphate dehydrogenase from *Leuconostoc mesenteroides* (Sigma-Aldrich). Reactions were performed similarly to spectroscopic assays using the PK/LDH system in 10-mm-path length quartz cuvettes in buffer O (50 mm HEPES-NaOH, pH 7.5, 200 mm NaCl). Final concentrations of d-glucose-6-phosphate dehydrogenase (G6P-DH) were 14.7 units/ml. 200 μm 3′-NADP from *in vitro* reactions or SMEs was added to the reaction after 2 min and then incubated for 10 min. To verify G6P-DH activity, 200 μm 2′-NADP (Roche Diagnostics GmbH) was added after the 10-min incubation period, and the reaction was allowed to proceed until all 2′-NADP was reduced to 2′-NADPH.

##### NMR Characterization of 3′-NADP

The concentration in our NMR experiments was 3 mg/ml. 3′-NADP purified from *in vitro* reactions was dissolved in 99.9% D_2_O and spectra were recorded at 25 °C using a Varian 500 NMR system spectrometer (Agilent). One dimensional NMR experiments were processed and analyzed using the TopSpin 3.2 software (Bruker BioSpin), heteronuclear single quantum coherence experiments were analyzed using the MestReNova 10.0 (MestreLab Research) software. Signal assignment of ^1^H NMR experiments (500 MHz, ^1^H-^1^H COSY, D_2_O) is: δ = 9.33 (s, 1H, H-N2), 9.15 (d, 1H, ^3^*J*_HH_ = 6.2 Hz, H-N6), 8.86 (d, 1H, ^3^*J*_HH_ = 8.2 Hz, H-N4), 8.47 (s, 1H, H-A8), 8.21 (m, 1H, H-N5), 8.16 (s, 1H, H-A2), 6.07 (m, 2H, N1′, H-A1′), 4.87 (m, 1H, H-A2′), 4.84–4.81 (m, 1H, H-A2′), 4.58 (m, 1H, H-A4′), 4.55 (m, 1H, H-N4′), 4.50 (“t”, 1H, ^3^*J*_HH_ ∼ 5.3 Hz, H-N2′), 4.44 (dd, ^3^*J*_HH_ = 5.0 Hz, H-N3′), 4.39–4.35 (m, 1H, H-N5′_a_), 4.31–4.22 (m, 3H, H-N5′_b_, H-A5′_a,b_) ppm.

Assignment of ^13^C NMR experiments (152.7 MHz, APT, HSQC, D_2_O) is: δ = 165.12 (C = O), 155.07 (C-A6), 152.36 (C-A2), 149.02 (C_q_), 145.69 (C-N4), 142.29 (C-N6), 139.82 (C-N2), 133.59 (C_q_), 128.57 (C-N5), 118.36 (C_q_), 99.97 (C-N1′), 87.00 (*J*_CP_ = 8.9 Hz, C-N4′), 86.18 (C-A1′), 83.21 (*J*_CP_ = 3.9 Hz, *J*_CP_ = 3.7 Hz, C-A4′), 77.51 (C-N2′), 73.90 (*J*_CP_ = 5.0 Hz, C-A3′), 73.33 (*J*_CP_ = 5.0 Hz, C-A2′), 70.61 (C-N3′), 65.34 (*J*_CP_ = 5.4 Hz, C-A5′), 64.89 (*J*_CP_ = 5.2 Hz, C-N5′) ppm.

Assignment of ^31^P NMR decoupling experiments (202.4 MHz, D_2_O) is: δ = 0.15 (s, P-A3′), −11.37 (d, *J*_PP_ = 20.3 Hz, P-5′), 11.66 (d, *J*_PP_ = 20.3 Hz, P-5′) ppm.

##### Spectrophotometric Substrate Comparison Assays

Substrate comparisons were performed with the PK/LDH coupled spectrophotometric assay similarly as described (29). Final assay conditions were 100 nm AvrRxo1 or AvrRxo1(D193N) (diluted in buffer G supplemented with 1 mg/ml BSA and 1 mm TCEP), 2.7 units/ml PK, and 4.0 units/ml LDH (Sigma-Aldrich) in 100 μl of buffer M containing 200 μm ATP and additional 400 μm PEP. Acceptor substrate candidates (NAD, NAAD, and UpA) or H_2_O as a negative control was added to 200 μm prior to starting the measurements. After 2 min, either AvrRxo1 or buffer G supplemented with 1 mg/ml BSA and 1 mm TCEP was added to the 10-mm-path length cuvette, and the reaction was incubated for 15 min. Subsequently, 200 μm NADH was added as substrate for LDH. The absorbance at 340 nm was constantly measured with a Jasco V-650 spectrophotometer.

##### Michaelis-Menten Kinetic Measurements of AvrRxo1 and AvrRxo1/2

Steady state kinetic measurements were performed with the PK/LDH coupled spectrophotometric assay similarly as described (29). Final assay conditions were 2.5 nm AvrRxo1, 2.7 units/ml PK, and 4.0 units/ml LDH (Sigma-Aldrich) in 550 μl of buffer M supplemented with 400 μm NADH and 1 mg/ml BSA. Note that NAD concentrations during titrations thereof had to be corrected by 56 μm due to a 1.8–1.9% ADP impurity of the ATP stock. For Michaelis-Menten kinetics in which Mg^2+^-ATP was titrated, a constant concentration of 4 mm NAD was included in the assay; when NAD or NAAD was titrated, a constant concentration of 3 mm Mg^2+^-ATP was used. Each NAD titration series of AvrRxo2 inhibition kinetics was performed in the absence or presence of 2.5, 5, 7.5, or 12.5 nm AvrRxo2. Here, Mg^2+^-ATP was also used at 3 mm. All reactions were carefully mixed in Eppendorf tubes and immediately transferred into quartz cuvettes (5-mm path length). Apparent reaction velocities were determined by monitoring the decrease in absorbance at 340 nm at 25 °C in a Jasco V-650 spectrophotometer using an automated, thermostated cell changer. Individual reaction velocities (Δ*A*/min) were determined using the kinetics analysis tool of the Spectra Manager software supplied by the manufacturer in time intervals of 145–756 s containing 5–36 data points. Those were converted to their apparent *k*_obs_ (s^−1^) values and fitted to a modified Michaelis-Menten equation, *k*_obs_ = ((*k*_cat_ × [*S*])/(*K_m_* + [*S*])) + *k*_basal_, using GraphPad Prism v.6.05 (GraphPad, La Jolla, CA). Note that a basal rate of ADP formation (*k*_basal_) independent from the titrated substrate was evident and thus included in the fit during NAD and NAAD titration experiments. This rate was determined to be 59 s^−1^.

## Author Contributions

F. S. and A. M. conceived and coordinated the study and wrote the paper. F. S., A. R., and D. E. designed, performed, and analyzed the experiments shown in Figs. 1, 2, and 4. F. S. and C. B. designed, performed, and analyzed the experiments shown in Fig. 3. F. S. and J. S. designed, performed, and analyzed the experiments shown in Figs. 5 and 6. All authors reviewed the results and approved the final version of the manuscript.

## Supplementary Material

Supplemental Data

## Footnotes

2 The abbreviations used are:

HR: hypersensitive response
PTI: pathogen-associated molecular pattern-triggered immunity
ETS: effector-triggered susceptibility
NAADP: nicotinic acid adenine dinucleotide phosphate
ETI: effector-triggered immunity
Avr: avirulence
UNAG: UDP-*N*-acetylglucosamine
SME: small metabolite extract
PK: pyruvate kinase
PEP: phosphoenolpyruvate
LDH: lactate dehydrogenase
UpA: uridine 5′ → 3′ adenine dinucleotide
AA: amino acid(s)
β-me: β-mercaptoethanol
CV: column volume
Ni-NTA: nickel-nitrilotriacetic acid
DTE: dithioerythritol
TEV: tobacco etch virus
mS: millisiemens
G6P-DH: d-glucose-6-phosphate dehydrogenase
TCEP: tris(2-carboxyethyl)phosphine)
ESI: electrospray ionization.
