# Supplementary material for: 3′-NADP and 3′-NAADP, Two Metabolites Formed by the Bacterial Type III Effector AvrRxo1
Source: J Biol Chem. 2016 Sep 12;291(44):22868–80. doi: 10.1074/jbc.M116.751297 (PMC5087710; doi:10.1074/jbc.M116.751297)
Supplement: Supplemental Data [file supp_291_44_22868__index.html]

3’-NADP and 3’-NAADP - Two Metabolites Formed by the Bacterial Type III Effector AvrRxo1 — 3′-NADP and 3′-NAADP, Two Metabolites Formed by the Bacterial Type III Effector AvrRxo1 — 3′-Phosphorylated NAD Derivatives — Supplemental Data 

# 3′-NADP and 3′-NAADP, Two Metabolites Formed by the Bacterial Type III Effector AvrRxo1

## Supplemental Data

- Supplemental data (.pdf, 986 KB) - supplemental data
