# Supplementary material for: 3′-NADP and 3′-NAADP, Two Metabolites Formed by the Bacterial Type III Effector AvrRxo1
Source: J Biol Chem. 2016 Sep 12;291(44):22868–80. doi: 10.1074/jbc.M116.751297 (PMC5087710; doi:10.1074/jbc.M116.751297)
Supplement: Supplemental Data [file 10.1074_M116.751297_jbc.M116.751297-1.pdf]

Supporting Information

Supporting Figures

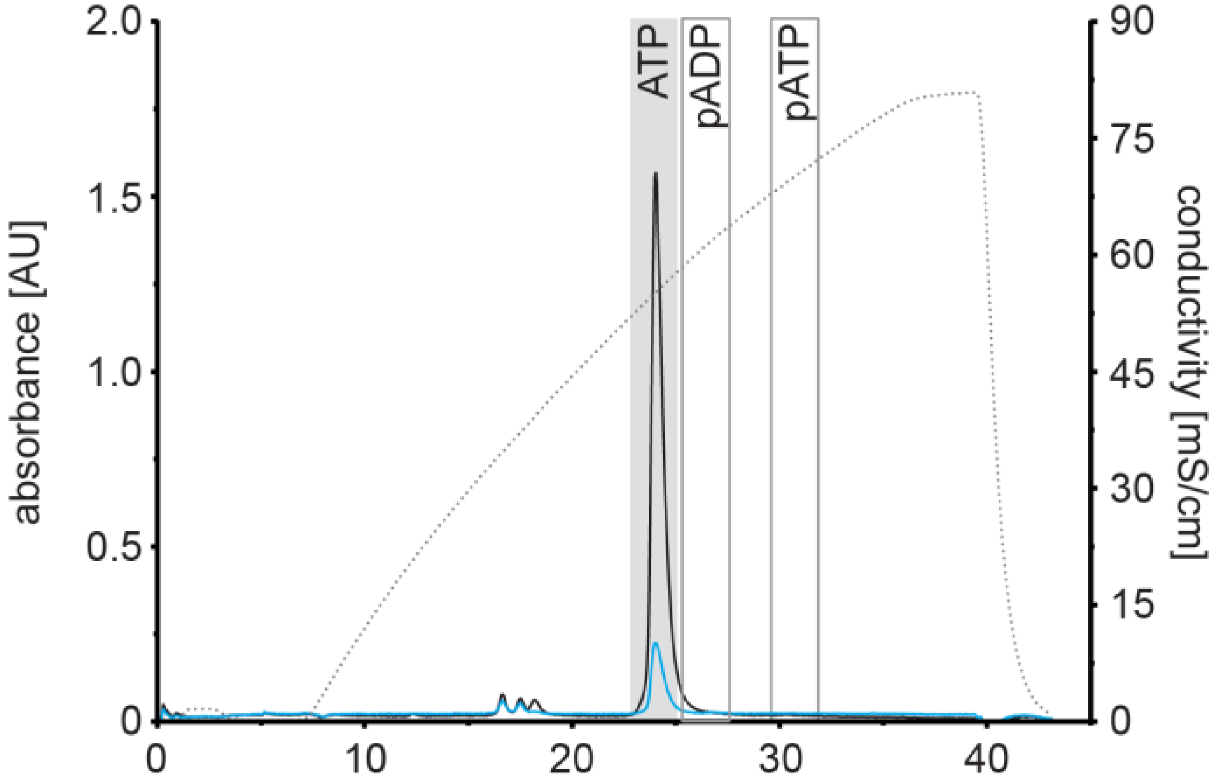

S1 Fig| Separation chromatogram of an ATP reaction mixture incubated with the catalytically impaired AvrRxo1(D193N) variant. Incubation of AvrRxo1(D193N) with 500 µM ATP for 5 h did neither result in accumulation of the phosphorylated ADP/ATP species observed when AvrRxo1 was used, nor in accumulation of ADP. AvrRxo1(D193N) is thus catalytically impaired and the phosphoryl transfer observed in AvrRxo1 / ATP incubations is specific for the enzyme. Traces shown are of A_{260} (black), A_{280} (blue), A_{340} (red) and eluate conductivity (dotted, gray line).

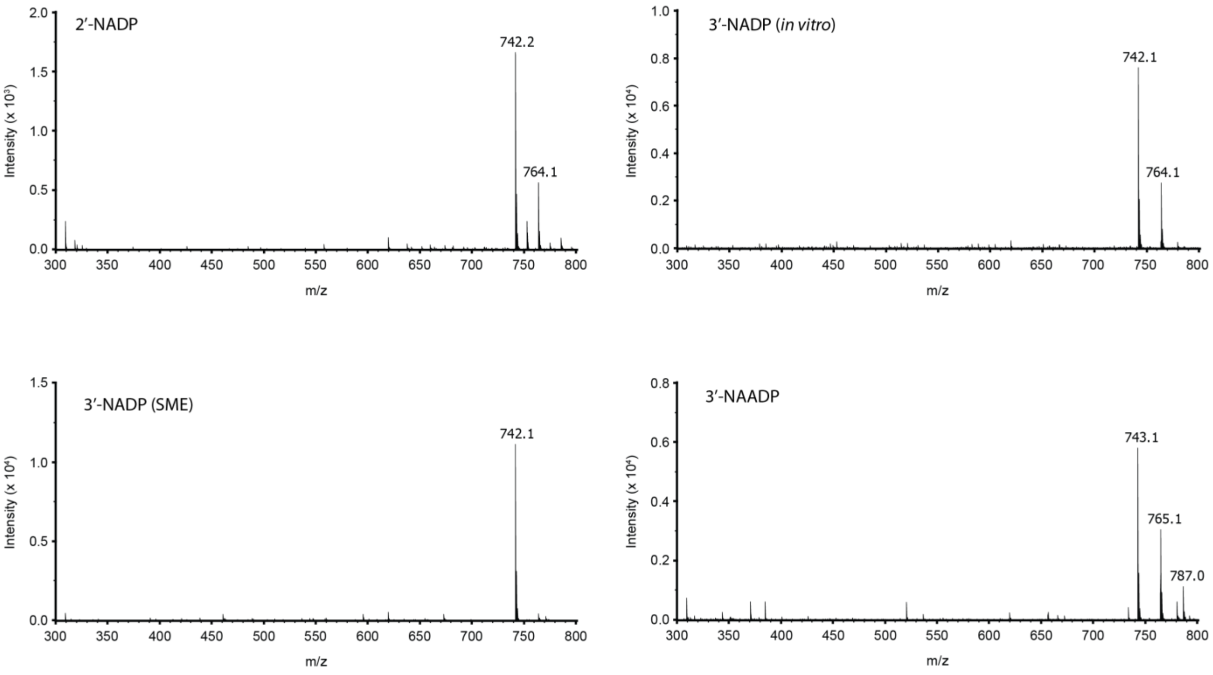

S2 Fig| ESI MS spectra of different NAD derivatives. Whole masses of 2’-NADP, 3’-NADP from *in vitro* reactions and 3’-NADP from small metabolite extracts are identical. The molecular mass of 3’-NAADP purified from *in vitro* reactions differs by 1 Da from that of 3’-NADP, implying that an additional proton was present on the molecule. Peaks differing in weight by 22 Da correspond to Na^+-adducts of the phosphorylated NAD / NAAD species.

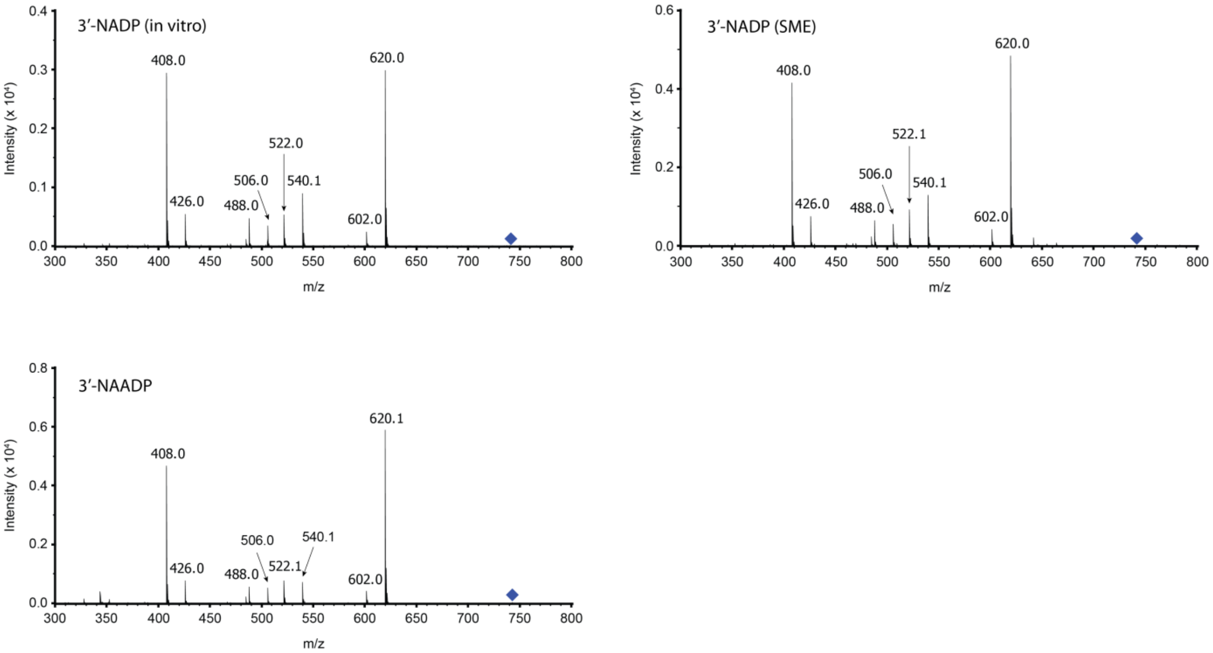

detected peak (Da)	408.0	426.0	488.0	506.0	522.0 and 522.1	540.1	602.0	620.0 and 620.1
Calc. exact mass (Da)	408.0	426.0	488.0	506.0	522.0	540.1	602.0	620.0
Structural formula	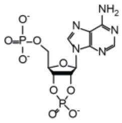	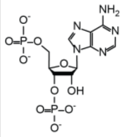 or 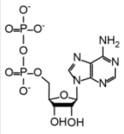	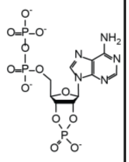	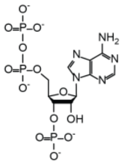	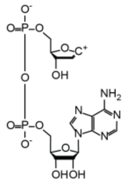	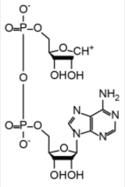	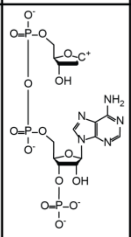 or 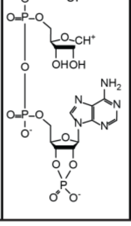	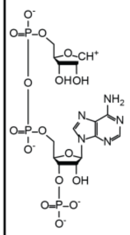

S3 Fig| ESI MS-MS spectra of 3'-NAADP and 3'-NADP from *in vivo* and *in vitro* with structural representations of detected fragments. Protons of the phosphates are not drawn in any of the structural formulas. Exact molecular masses were calculated with associated protons. Abbreviations: Calc. exact mass = calculated exact mass (theoretical mass of compound composed of only the highest abundant isotopes); Da = Dalton. The blue diamond in ESI spectra indicates the species selected for fragmentation.

A

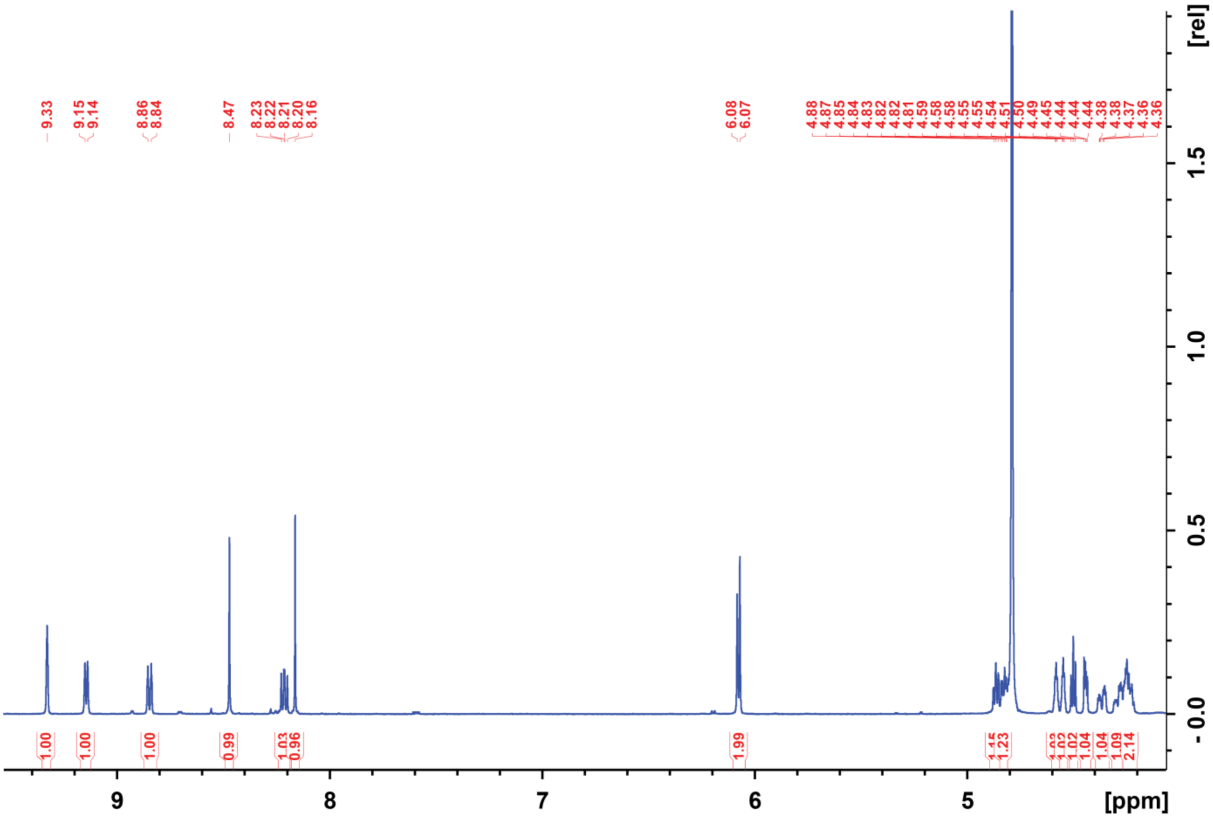
9.33
9.15
9.14
8.86
8.84
8.47
8.23
8.22
8.21
8.20
8.16
6.08
6.07
1.00
1.00
1.00
0.99
1.03
0.96
1.99
1.23
1.04
1.04
1.09
2.14
[rel]
[ppm]

B

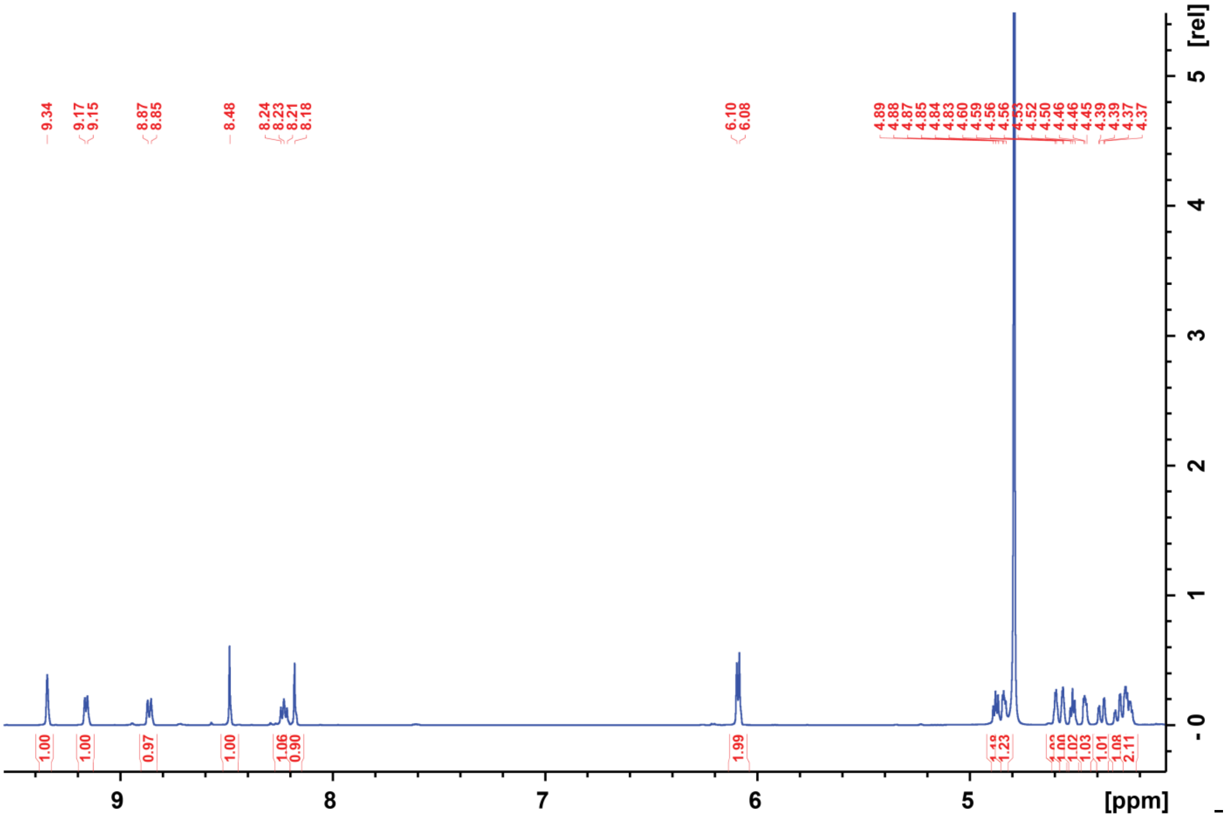
9.34
9.17
9.15
8.87
8.85
8.48
8.24
8.23
8.21
8.18
6.10
6.08
1.00
1.00
0.97
1.00
1.06
0.90
1.99
1.23
1.03
1.01
1.08
2.11
[rel]
[ppm]

C

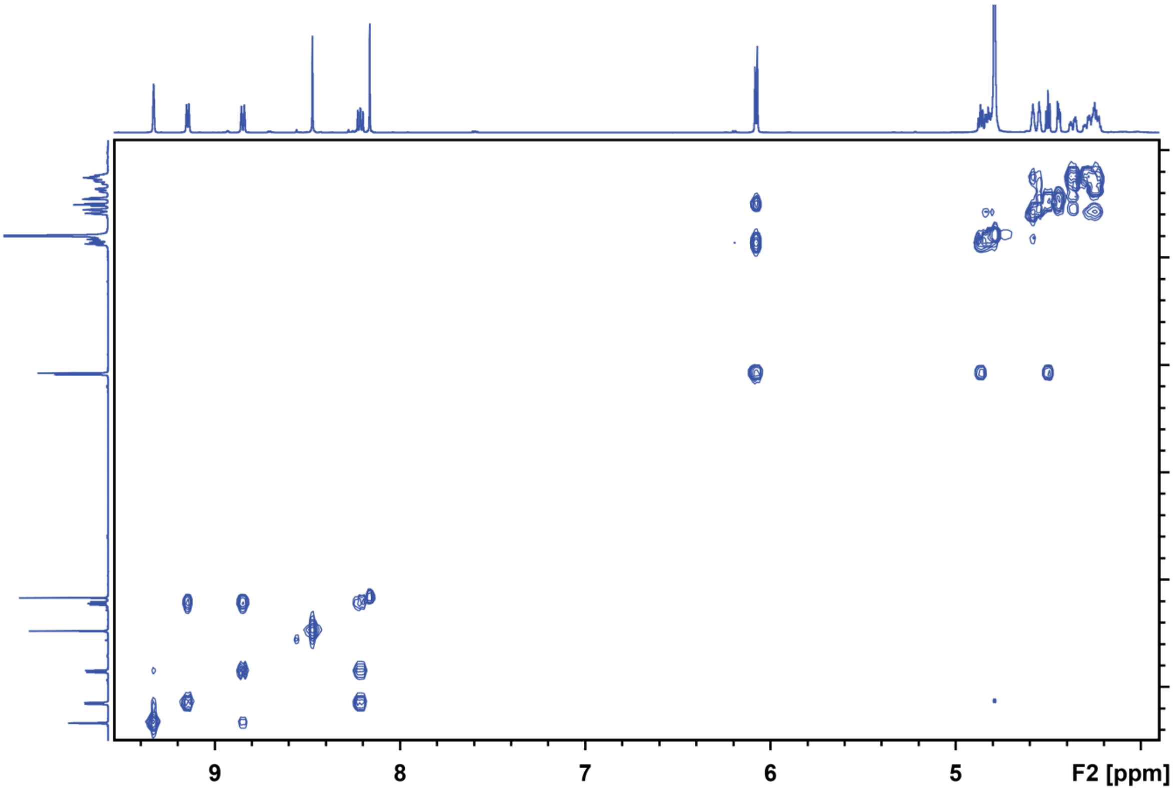
F1 [ppm]
F2 [ppm]
5
6
7
8
9
9
8
7
6
5

D

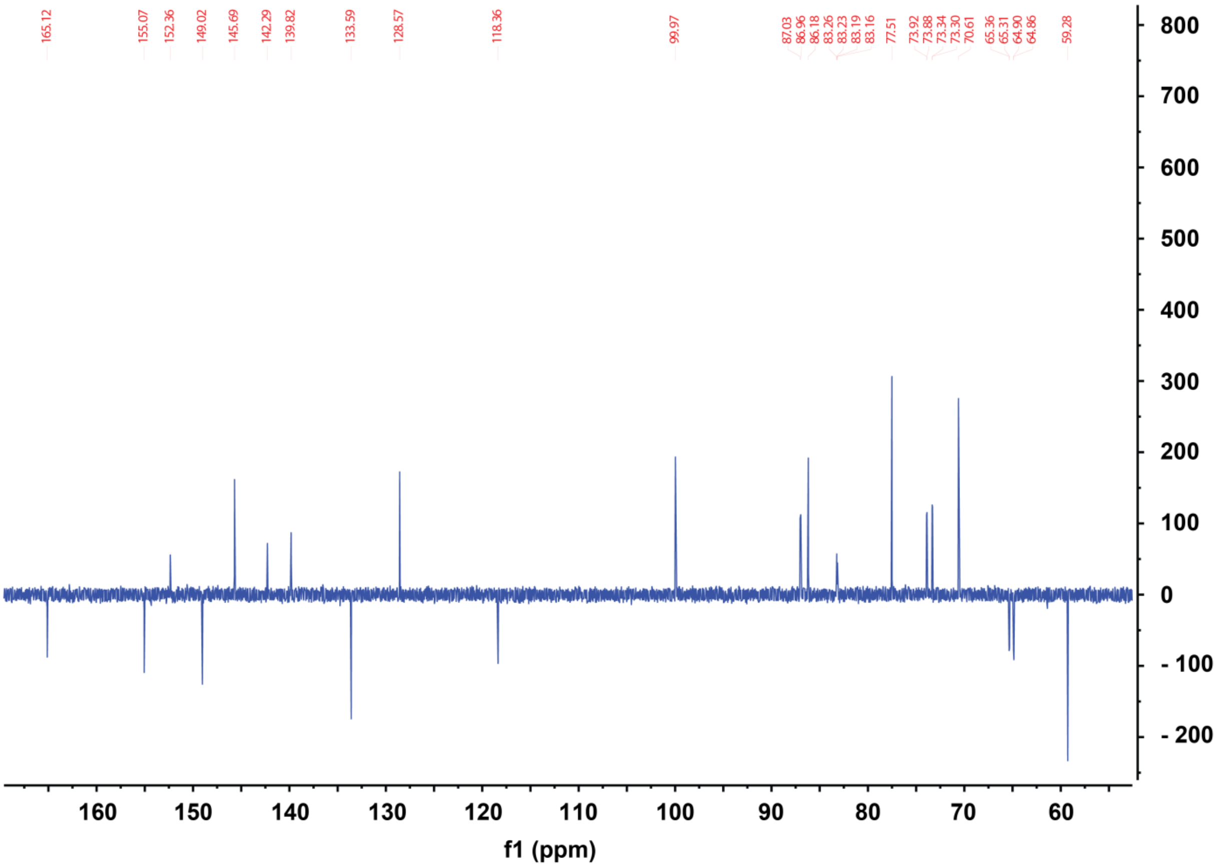
165.12
155.07
152.36
149.02
145.69
142.29
139.82
133.59
128.57
118.36
99.97
87.03
86.96
86.18
83.26
83.23
83.19
83.16
77.51
73.92
73.88
73.34
73.30
70.61
65.36
65.31
64.90
64.86
59.28
800
700
600
500
400
300
200
100
0
- 100
- 200
160
150
140
130
120
110
100
90
80
70
60
f1 (ppm)

E

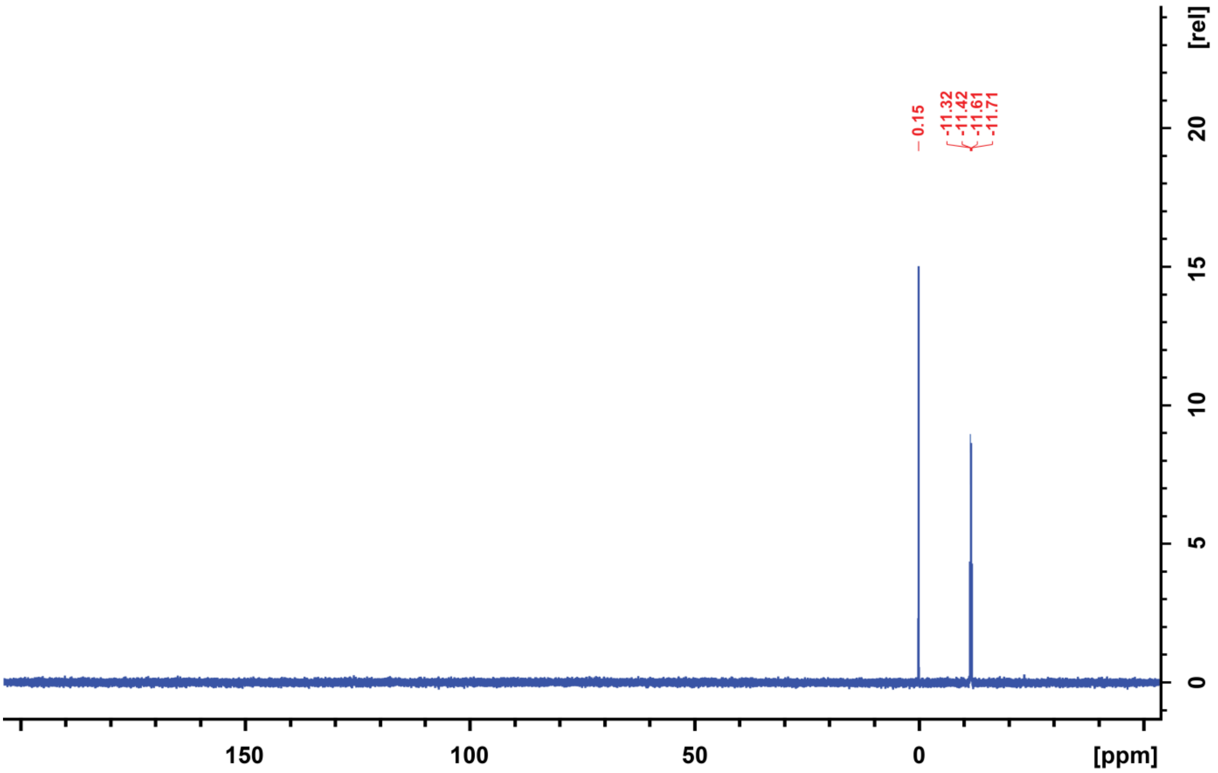

S4 Fig| NMR spectra of 3’-NADP in D_2O. (A) ^{1}H-NMR (500 MHz). (B) ^{1}H-^{31}P-decoupled NMR (500 MHz). (C) ^{1}H-^{1}H-COSY NMR (500 MHz). (D) ^{13}C-APT NMR (125.7 MHz). (E) ^{31}P-NMR (202.4 MHz).
